# High-Density Gold Nanoparticles Implanted on Mg/Fe LDH Nanoflowers Assisted Lateral Flow Immuno-Dipstick Assay for Visual Detection of Human Epididymal Protein 4

**DOI:** 10.3390/bios12100797

**Published:** 2022-09-27

**Authors:** Hao Liu, Mei-Xia Wu, Shou-Nian Ding

**Affiliations:** 1 Jiangsu Province Hi-Tech Key Laboratory for Bio-Medical Research, School of Chemistry and Chemical Engineering, Southeast University, Nanjing 211189, China; 2 Lianshui Peoples Hospital, Huaian 223400, China

**Keywords:** layered double hydroxide nanoflowers, gold nanoparticles, human epididymal protein 4, lateral flow immuno-dipstick assay

## Abstract

The timelier and more accurate the diagnosis of the disease, the higher the patient’s survival rate. Human epididymal protein 4 (HE4) has great significance as a biomarker of concern for reflecting ovarian cancer. Herein, we prepared a novel optical label that can be used in lateral-flow immuno-dipstick assay (LFIA) for sensitive visual detection of HE4 by implanting hydrophobic gold nanoparticles (Au NPs) at high density in Mg/Fe LDH nanoflowers (MF NFs). MF NFs with large specific surface area, high porosity, abundant active binding sites, and stable structure were employed for the first time as templates to directly anchor Au NPs in the organic phase. After simple modification with an optimized amount of branched polyethyleneimine, not only could MF@Au NFs be dispersed in the aqueous phase, but also amino functional groups were introduced on its surface to facilitate subsequent antibody coupling steps. The limit of detection reaches 50 pM with a detection range of 50 to 1000 pM. This work initially explored how MF NFs can be used to load signal labels with ideal stability and signal amplification capabilities, which greatly improves the practicability of LFIA and highlights its important role in the field of rapid diagnostics.

## 1. Introduction

Ovarian cancer is one of the three most common malignant tumors in the female reproductive system [1,2]. Most ovarian cancer patients are in the middle and late stages when they are first diagnosed and thus lose the best time for treatment [3,4,5]. Therefore, early diagnosis has important clinical significance for the treatment and prognosis of ovarian cancer. As a new tumor marker discovered in recent years, human epididymal protein 4 (HE4) has been studied by many researchers in various respects [6,7,8,9,10,11,12]. HE4 is not expressed in normal ovarian tissue but is abundantly expressed in ovarian cancer tissue. Ingegerd Hellström et al. found that HE4 was overexpressed in epithelial ovarian cancer in 2003, demonstrating for the first time that HE4 protein has potential as a biomarker for ovarian cancer [13]. As we all know, early detection and early treatment is the mainstream idea of current diagnosis, which will help reduce the morbidity and mortality of the disease and ultimately improve the quality of life of patients. Although traditional methods such as enzyme-linked immunosorbent assay and quantitative real-time PCR can help solve the early diagnosis of diseases, they are limited by expensive and complicated laboratory equipment and well-trained operators [14].

Lateral flow immuno-dipstick assay (LFIA), as a star product in point-of-care devices, has already played an important role in medical diagnosis, food analysis, and environmental monitoring [15,16,17]. Gold nanoparticles (Au NPs) and latex microbeads are the main tags currently used to construct LFIA. Among them, Au NPs are favored by researchers in photonics, catalysis, and bio-nanotechnology due to their inherently superior physicochemical properties and easy functionalization [18,19,20,21]. Notably, since the localized surface plasmon resonance effect is very sensitive to particle size, structure, composition, and interparticle distance, Au NPs have been further utilized in optical detection platforms. This colorimetric assay based on a single gold nanoparticle as a signal reporter molecule usually suffers from low sensitivity in low-abundance biomarker systems, which seriously hinders its application in early diagnosis [22]. In addition, the currently widely used optical labels are usually water-phase synthesized Au NPs, which have inherent disadvantages such as wide size distribution and insufficient stability. These shortcomings can be addressed by the oil-phase synthesized Au NPs because these nanocrystals have the advantages of controllable particle size and uniform morphology, and the surface of the nanoparticles is capped by alkyl ligands with stable surface chemical properties [23,24]. Several studies have demonstrated that template-based loading is an effective strategy to obtain composites with good size, shape, and configuration [25,26,27]. The three-dimensional incorporation of gold units into the template is highly desirable, which allows for a uniform distribution of Au NPs and is beneficial for preserving the plasmonic properties of individual Au NPs.

**Figure 1:**
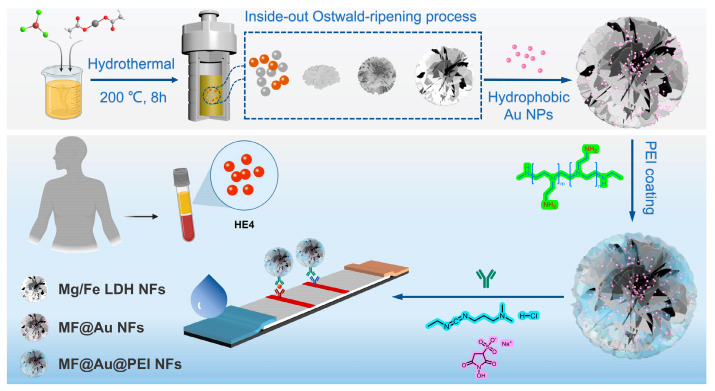
Schematic diagram of the preparation of high-density Au NPs-implanted Mg/Fe-LDH nanoflowers and detection process for MF@Au@PEI−LFIA.

Layered double hydroxides (LDHs) in the form of anionic clays have attracted increasing attention from researchers due to their layered structure, high surface area, porous structure, and interlayer ion exchange [28]. Among them, spherical LDH nanoparticles with porous structure have attracted much attention due to their structural stability and high surface area. Mahfuza Mubarak et al. synthesized Mg/Fe-LDH hollow nanospheres with high specific surface areas by a simple ethylene glycol-mediated thermal method using only two metal precursors, Mg^2+^ and Fe^3+^ [29]. In this work, Mg/Fe LDH nanoflowers (MF NFs) were used for the first time as templates for loading hydrophobic Au NPs due to their advantages of large surface area, high porosity, abundant active binding sites, and stable structure [30,31,32]. As illustrated in Figure 1, a large amount of hydrophobic Au NPs were loaded into MF NFs through thiol-metal covalent bonds. The outer layer of MF@Au nanocomposites consists of branched polyethyleneimine (PEI), which can not only serve as a hydrophilic modification layer but also introduce amino functional groups to facilitate subsequent functional derivatization. Finally, a new type of high-density Au NP-incorporated MF NFs were successfully prepared. In the MF@Au@PEI−LFIA, MF@Au@PEI NFs containing amino functional groups on the surface were carboxylated by succinic anhydride and combined with anti-HE4 labeled antibodies to prepare the bioconjugates. Subsequently, different concentrations of HE4 antigen are combined with bioconjugates and applied to the LFIA strip. Qualitative results can be measured by using the naked eye within 15 min. MF@Au@PEI−LFIA exhibits higher sensitivity and a broader linear region compared with those of colloidal gold test strips.

## 2. Materials and Methods

### 2.1. Chemicals and Biological Reagents

Iron (III) chloride hexahydrate, magnesium acetate tetrahydrate, tetrachloroauric (III) acid tetrahydrate, glutaraldehyde, ethylene glycol, anhydrous ethanol, oleylamine (OLM), and Triton X-100 were purchased from Sinopharm Chemical Reagent Co., Ltd. Branched polyethyleneimine (PEI, MW 25000) was purchased from Sigma-Aldrich (Burlington, MA, USA). Bovine serum albumin (BSA), trimethoxysilylpropanethiol (3-MPTMS), 1-ethyl-3-[3-dimethylaminopropyl] carbodiimide hydrochloride (EDC·HCl), and sodium 1-hydroxy-2,5-dioxopyrrolidine-3-sulfonate (Sulfo-NHS) were purchased from Energy Chemical Co., Ltd. (Bingham, Nottinghamshire, UK). All chemicals were used as received without purification. Phosphate buffer solution (PBS, 0.01 M, pH 7.4), MES buffer solution (0.01 M, pH = 6.0), and HEPES buffer solution (0.01 M, pH = 7.4) were freshly prepared before use. The Anti-HE4 monoclonal antibody (Ab_1_&Ab_2_), AFP, CA199, and CEA were purchased from Shanghai Linc-Bio Science Co., LTD (Shanghai, China). Goat anti-mouse IgG antibody, sample pads, polyvinyl chloride (PVC) substrate, nitrocellulose (NC) membranes, absorbent pads, and plastic adhesive cards were purchased from Shanghai Joey Biotechnology Co. Ltd. (Shanghai, China).

### 2.2. Characterization

^13^C CP/MAS NMR spectra were obtained using a Bruker ASCEND^TM^ 400WB spectrometer at 400 MHz and reported as parts per million (ppm). UV-vis absorbance spectra were obtained on a Shimadzu UV 2600 spectropolarimeter (Japan). Scanning electron microscopy (SEM) measurements were carried out under field emission scanning electron microscopy (Thermo Scientific, Waltham, MA, USA) operated at 20 kV. Transmission electron microscopy (TEM) measurements were carried out under a field-emission high-resolution transmission electron microscopy Talos F200X (Thermo Scientific, Waltham, MA, USA). Fourier transform infrared (FT-IR) spectrum was collected from a Nicolet 5700 (Thermo Nicolet Corporation, Waltham, MA, USA) IR spectrometer in the range of 4000–400 cm^−1^. Powder X-ray diffraction (XRD) analysis was carried out using Rigaku Ultima IV multifunctional horizontal X-ray diffractometer. X-ray photoelectron spectroscopy (XPS) studies were performed using the XPS-2 (PreVac, Poland) system. Dynamic light scattering (DLS) and ζ-potential data were collected from NanoBrook 90 Plus PALS.

### 2.3. Preparation of Mg/Fe LDH Nanoflowers

Mg/Fe LDH nanoflowers (MF NFs) were synthesized according to a previous study with some minor modifications [29]. Briefly, Mg (OAc)_2_·4H_2_O (3.0 g, 14.1 mmol) was put into a three-necked flask containing 120 mL of ethylene glycol and ultrasonically dissolved quickly. Then, FeCl_3_·6H_2_O (285 mg, 1.05 mmol) dissolved in 30 mL of ethylene glycol was added to the flask through a dropping funnel, and the mixture was allowed to form a homogeneous yellow clear solution with magnetic stirring for 4 h. Afterwards, the solution was transferred to a Teflon-lined autoclave and heated at 200 °C for 8 h. The final product was cooled to room temperature and then washed three times with ethanol and water. The yellowish MF NFs were obtained after vacuum drying at 60 °C overnight.

### 2.4. Synthesis of OLM Capped Gold Nanoparticles (Au NPs)

Au NPs were synthesized by rapidly injecting gold precursor into a pre-heated surfactant solution [24]. Briefly, 10 mL of OLM was injected into a 25 mL three-neck flask and refluxed at 150 °C under an N_2_ atmosphere. Then, HAuCl_4_·4H_2_O (0.2472 g, 0.6 mmol) was dissolved in OLM (2 mL), and it was quickly injected into the flask to continue the heating reaction for 2 h to obtain monodisperse Au NPs.

### 2.5. Preparation of MF@Au@PEI Nanoflowers

Firstly, MF NFs needed to be modified with sulfhydryl functional groups. MF NFs (50 mg) were dispersed in absolute ethanol by ultrasonication, and then NH_3_·H_2_O (500 μL) and MPTMS (500 μL) were added, followed by vigorous stirring at 25 °C for 6 h. The sulfhydryl-terminated MF NFs were harvested by centrifugation, washed thoroughly with ethanol and H_2_O, and dispersed in 50 mL of chloroform. Secondly, excess Au NPs chloroform dispersion was added to the above sulfhydryl-terminated MF NFs chloroform dispersion, and a clear and transparent solution was obtained via ultrasonically treatment for 20 min. The complexes were washed twice with chloroform to remove unlinked AuNPs. The MF@Au NFs were properly dried in airflow and then dispersed in a 20 mL chloroform solution containing PEI (0.2 mg/mL) under ultrasonication conditions for 20 min. After centrifugation and washing, the MF@Au@PEI NFs can be directly dispersed in PBS solution and stored at 4 °C for later use.

### 2.6. Preparation of Ab_2_-MF@Au@PEI

The specific preparation process is roughly the same as that reported in the previous literature [33]. Briefly, MF@Au@PEI NFs with amino functional groups on the surface were dispersed in water at a concentration of 1 mg/mL, and succinic anhydride solution (5 mg/mL) was added and stirred for 4 h to modify MF@Au@PEI with carboxyl groups. After that, it was washed several times with ethanol and water and dispersed in MES buffer solution (0.01 M, pH = 6.0) for further use. One milligram of carboxyl-modified MF@Au@PEI NFs were ultrasonically dispersed into 0.5 mL of MES buffer solution, 4 mg of EDC and 6 mg of Sulfo-NHS were added in sequence, and the reaction was shaken for 15 min. The activated MF@Au@PEI NFs were washed with water twice and redispersed in 0.5 mL of HEPES buffer (0.01 M, pH = 7.4). Seventy-five micrograms of Ab_2_ (0.5 mL, 0.15 mg/mL) was added to the above solution and incubated for 2 h at room temperature with shaking. Finally, Ab_2_-MF@Au@PEI bioconjugates were collected by centrifugation, washed with HEPES buffer, and redispersed in 1 mL of HEPES buffer solution (0.01 M, pH = 7.4, containing 1% BSA) to form a storage dispersion for later use.

### 2.7. Fabrication of the MF@Au@PEI−LFIA Test Strips

LFIA test strips were prepared by our previous reported literature [33,34]. The MF@Au@PEI−LFIA consists of six parts, including polyvinyl chloride (PVC) substrate, nitrocellulose (NC) membrane, absorbent paper, sample pad, HE4 test line, and control line. The sample pad was pretreated with PBS (0.01 M, pH = 7.4) containing 1% Triton X-100 and 2% NaCl solution and dried overnight at room temperature. The capture antibody (Ab_1_, 2 mg/mL) and goat anti-mouse IgG antibody (2 mg/mL) were dispersed in the test line and control line on the NC membrane, respectively. After that, the absorbent-pad-modified NC membrane and the pretreated sample pad were assembled in sequence on the PVC adhesive backing. Then, the assembled strips were cut into 4 mm wide pieces. Finally, the prepared test strips were sealed and stored in a light-proof box.

### 2.8. Detection of HE4 with the MF@Au@PEI−LFIA

Different concentrations of 60 μL HE4 standard target solutions (0, 5, 10, 20, 50, 100, 200, 400, 800, and 1000 pM) were premixed with Ab_2_-MF@Au@PEI NFs (40 μL), respectively. Afterward, the mixtures were applied to the sample pad. Each concentration was detected three times. After 15 min, qualitative results could be obtained by observing the red bands on the strips. An Apple mobile phone camera was used to take pictures for qualitative measurements, and ImageJ software was used to digitally process the images for T-line and C-line intensities. To compensate for potential intensity variations caused by acquisition conditions such as lighting and camera settings, background subtraction was required, and this work used the ratio of T-line intensity to C-line intensity (T/C) to quantify the signal [35,36].

## 3. Results and Discussion

### 3.1. Synthesis and Characterization of the MF@Au@PEI NFs

**Figure 2:**
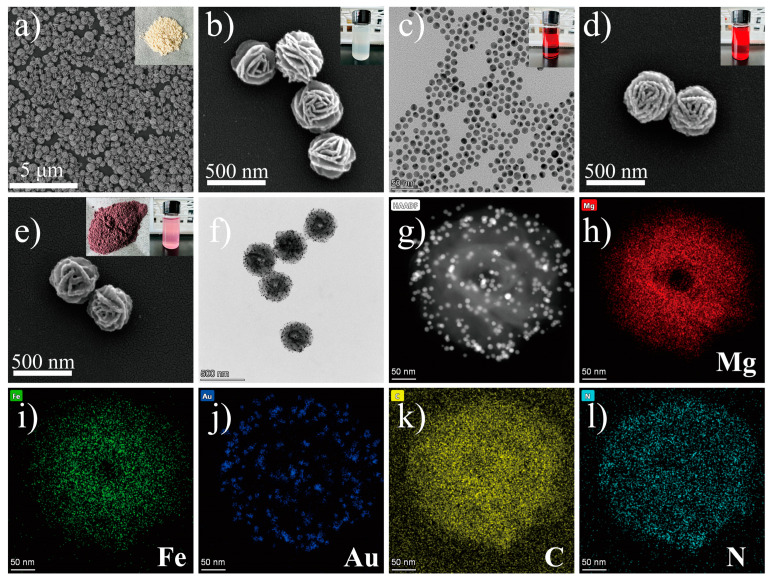
(**a**,**b**) SEM and TEM images of Mg/Fe LDH NFs. (The two insets are MF NFs solid powder and the state dispersed in H_2_O.) (**c**) OLM capped Au NPs. (The inset is the state dispersed in CHCl_3_.) (**d**) SEM images of MF@Au NFs. (The inset is the state dispersed in CHCl_3._) (**e**,**f**) SEM and TEM images of MF@Au@PEI NFs. (The two insets are the MF@Au@PEI solid powder and the state dispersed in H_2_O.) (**g**–**l**) HAADF STEM image and EDS mapping images of MF@Au@PEI NFs.

**Figure 3:**
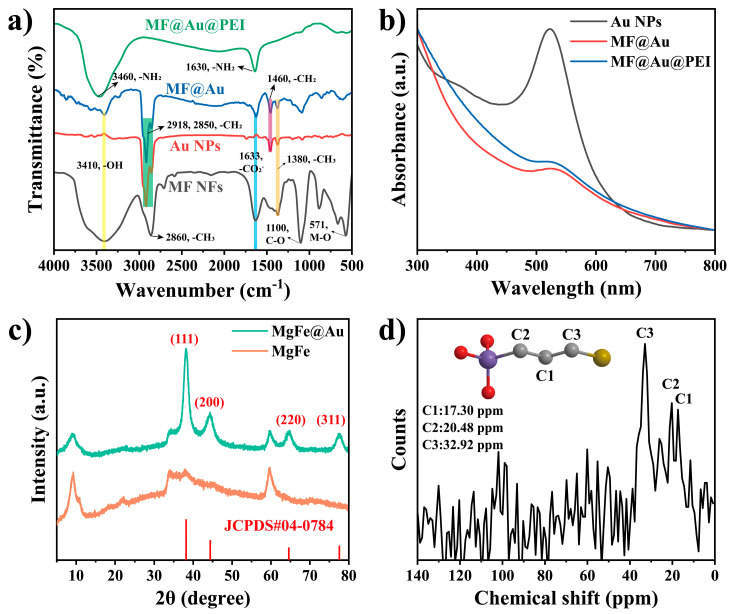
(**a**) FT-IR spectra of MF NFs, Au NPs, MF@Au NFs, and MF@Au@PEI NFs. (**b**) UV-vis absorption spectra of Au NPs, MF@Au NFs, and MF@Au@PEI NFs. (**c**) The XRD patterns of MF and MF@Au NFs. (The vertical red solid lines stand for diffractions according to JCPDS#04-0784.s) (**d**) Solid-state ^13^C CP/MAS NMR spectrum of SH-MF NFs. (The inset is the schematic diagrams of 3-MPTMS.)

**Figure 4:**
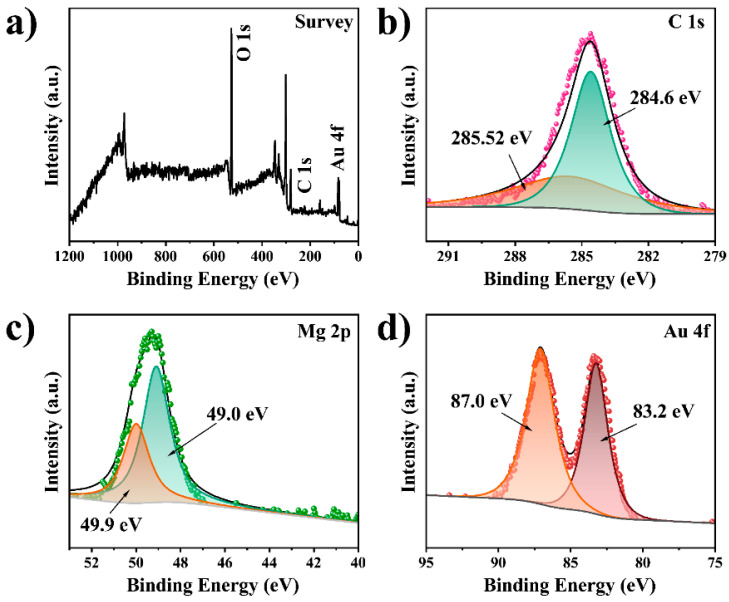
(**a**) XPS survey spectra, and high resolution (**b**) C 1s, (**c**) Mg 2p, and (**d**) Au 4f spectra of MF@Au NFs.

Mg/Fe LDH NFs were synthesized by using magnesium acetate and ferric chloride as precursors and ethylene glycol as a solvent in an autoclave. Figure 2a,b and Figure S3a are typical images of the as-prepared MF NFs. These flower-like nanospheres consist of many nanosheets with a thickness of about 10 nm interconnected to form highly open structures with dimensions of 380–400 nm (Figure S5a in Supplementary Materials). The growth mechanism of MF can be explained by the so-called inside-out Ostwald maturation process proposed by Song et al. [37,38,39]. Firstly, magnesium ions and iron ions coagulate and self-assemble into solid flower-like spheres. Then, nanosheets on the spherical shell begin to grow, while the inner core begins to gradually form voids, and finally, 3D hierarchical flower-like hollow nanospheres are obtained. After the sulfhydryl functional group modification of MF, OLM-capped AuNPs with uniform size (10 nm, Figure 2c, Figures S1 and S5d) prepared by reducing chloroauric acid in the organic phase were reacted with SH-MF ultrasonically for 20 min in chloroform solution to obtain MF@Au NFs. It can be seen from Figure 2d and Figure S3b that there are many prominent Au NPs on the surface of MF NFs, which proves that the Au NPs are abundantly loaded into the MF NFs. Likewise, the high density of Au NPs bound to MF can be more clearly seen by TEM (Figure 2f and Figure S3c,d). It can be seen from Figure 2e that after the MF@Au NFs are coated with PEI hydrophilic modification, the Au NPs on its surface are wrapped. Energy-dispersive X-ray spectroscopy (EDS) elemental mapping, nuclear magnetic resonance (NMR), and Fourier transform infrared (FT-IR) techniques were employed to investigate the composition of MF@Au@PEI NFs. As shown in Figure 2g–l and Figure S4, MF@Au@PEI NFs are composed of Mg, Fe, Au, C, and N elements, which can demonstrate the successful preparation of the MF@Au@PEI composite structure. In the FT-IR spectra (Figure 3a), the MF NFs prepared in this work are in good agreement with the characteristics of LDH-type materials reported in the literature [29,40]. The characteristic peaks located at 2918 cm^−1^ (stretching vibration of -CH_2_), 2850 cm^−1^ (stretching vibration of -CH_2_), 1460 cm^−1^ (stretching vibration of -CH_2_), and 1380 cm^−1^ (stretching vibration of -CH_2_) indicate the successful preparation of OLM-capped Au NPs. The curves of MF@Au are in good agreement with OLM-capped Au NPs, which indicates the formation of MF@Au composites. After PEI coating, the characteristic peaks at 3460 cm^−1^, and 1630 cm^−1^ indicate the presence of amino functional groups. Not only did the MF@Au@PEI NFs show good hydrophilicity, but their surface was also modified with amino groups, which facilitated the subsequent antibody conjugation procedures. Figure 3b shows the UV-vis absorption data that the absorption peaks of the three are all around 520 nm. The X-ray powder diffraction (XRD) patterns of the MF NFs and MF@Au are shown in Figure 3c. The MF NFs prepared in this work have five main peaks at 9.26°, 22.12°, 34.1°, 42.3°, and 59.7°, corresponding to the (003), (006), (012), (015) and (110) planes, respectively [29]. The Au diffraction peaks in the MF@Au composite are well preserved, and the peak shape corresponds to the standard card one-to-one (JCPDS#04–0784), showing the cubic structure of Au NPs. These indicate that the crystal structure of the oil-phase AuNPs prepared in this work were not affected during the entire assembly process. In the ^13^C CP/MAS NMR spectrum of SH-MF NFs (Figure 3d), three peaks at 17.30, 20.48, and 32.92 ppm can be assigned to methylene carbons originated from the MPTMS, which indicates that thiol functional groups have been modified onto the MF NFs. The ζ-potential results of MF NFs, hydrophobic Au NPs, MF@Au, and MF@Au@PEI are shown in Figure S5f. The ζ-potential of MF NFs in the water phase is negative (−54.92 mV) due to the large number of hydroxyl functional groups. The change in the ζ-potential value of MF@Au indicates that Au NPs are loaded in MF NFs, which turns the ζ-potential into a positive charge (53.26 mV). The surface composition and chemical state of the MF@Au NFs were investigated by X-ray photoelectron spectroscopy (XPS). The MF NFs are mainly composed of C, O, Mg, and Fe elements (Figure S6), which is consistent with the XPS data reported in the literature [29]. As shown in Figure 4a, MF@Au NFs are mainly composed of C, O, Mg, and Au elements. High-resolution XPS spectra were recorded for C 1s, Mg 2p, and Au 4f, as shown in Figure 4b–d. The C 1s spectrum consists of two components with binding energies around 284.6 eV and 285.5 eV, corresponding to C–C and C–OH bonds, respectively [29,41]. The Mg 2p spectrum has two peaks at about 49.0 and 49.9 eV, which can be ascribed to Mg–OH and Mg–O bonds, respectively [29,42]. Figure 4d shows the XPS spectrum of Au 4f, and the curve fitting analysis shows that Au 4f_7/2_ and Au 4f_5/2_ have two peaks at 83.2 eV and 87.0 eV, respectively. The Au 4f doublet of the sample is split to 3.8 eV, indicating that Au exists only in the metallic state [43]. The above characterization results all prove the successful preparation of MF@Au@PEI NFs.

### 3.2. Performance of MF@Au@PEI−LFIA for HE4 Detection

**Figure 5:**
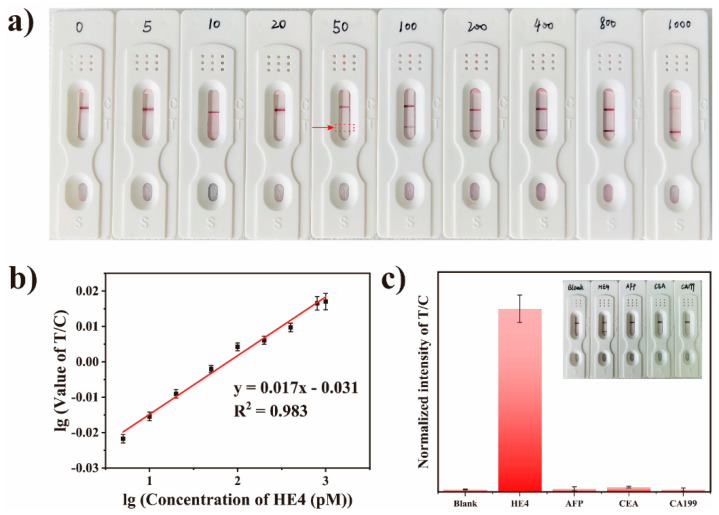
(**a**) Photograph of the MF@Au@PEI−LFIA when detecting different concentrations of HE4 (0, 5, 10, 20, 50, 100, 200, 400, 800, and 1000 pM). (**b**) Linear response of MF@Au@PEI−LFIA for detection of HE4 in the concentration range of 50−1000 pM. (**c**) Specificity of MF@Au@PEI−LFIA for different biomarkers (AFP, CEA, CA199).

Before implementing the MF@Au@PEI−LFIA, the optimization of the concentrations of PEI is very critical to achieve the best detection effect. Four different concentrations of PEI (5, 1, 0.5, 0.2 mg/mL) were used to coat MF@Au NFs. As shown in Figure S7, when the concentration exceeds 0.2 mg/mL, the MF@Au NFs experience different degrees of coagulation. We believe that the excessive PEI coating may lead to the mutual adhesion of the NFs, and the sedimentation occurs under the action of gravity. Therefore, the PEI concentration used in this work was 0.2 mg/mL. Various concentrations of HE4 antigen standard solutions were applied to analyze the sensitivity of the MF@Au@PEI−LFIA. The antigen standard solutions of different concentrations were premixed with Ab_2_-MF@Au@PEI NFs and then added to the sample pads. The qualitative model of MF@Au@PEI−LFIA is based on visual observation of the line color. The test result images of the test strip after 15 min are shown in Figure 5a. All test strips showed a control line, which proved that the test results are valid. The results indicated that the limit of detection using the naked eye observation was approximately 50 pM for HE4. The signal intensity values of the test line and the control line were obtained by taking advantage of ImageJ software. As shown in Figure 5b, the linear relationship of HE4 between the T line intensity value/C line intensity value and the antigen concentrations is y = 0.013x − 0.024 (R^2^ = 0.983). Clinical medicine considers the concentration of HE4 to be positive for ovarian cancer when it exceeds 70 pM [44]. As a comparison, we detected HE4 standard samples at various concentrations using traditional citrate-capped Au NPs as optical labels. As shown in Figure S8, when the concentration of HE4 was 800 pM, the T line still did not show a red band, indicating that the traditional citrate-capped Au NPs as optical labels had low detection sensitivity for HE4. Meanwhile, this result verifies the optical signal amplification of MF@Au@PEI as an optical label in antigen immune response. In addition, the assessment of three biomarkers (AFP, CEA, and CA199) at a concentration of 1 μg/mL verified the specificity of MF@Au@PEI−LFIA. The experimental results are shown in Figure 5c, and none of the other three biomarkers can produce a red band on the T line, except for HE4, which proved that MF@Au@PEI−LFIA has good selectivity.

## 4. Conclusions

In summary, we prepared a novel optical label by efficiently implanting hydrophobic Au NPs into Mg/Fe LDH nanoflowers. Compared with traditional colloidal gold nanotags, the incorporation of high-density Au NPs greatly enhanced the optical signal intensity of each carrier, and the detection sensitivity of HE4 was greatly improved. Under optimal experimental conditions, the limit of detection for HE4 was 50 pM with a detection range of 50 to 1000 pM. The study found that MF@Au@PEI−LFIA is highly selective for HE4 and has little mutual interference with other biomarkers (AFP, CA199, CEA). This work initially explores how Mg/Fe LDH NFs can be used to load signal labels with desirable stability and signal amplification capabilities, which greatly broadens the practicability of LFIA and highlights its important role in rapid diagnosis.

## Data Availability

Not applicable.

## Supplementary Materials

The following supporting information can be downloaded at: https://www.mdpi.com/article/10.3390/bios12100797/s1, Figure S1. TEM images of OLM capped Au NPs. Figure S2. TEM images of Citrate capped Au NPs. Figure S3. (a). TEM image of MF NFs. (b). SEM image of MF@Au NFs. (c–d). TEM image of MF@Au@PEI NFs. Figure S4. The EDS spectrum of MF@Au@PEI NFs. Figure S5. (a–e) DLS data of hydrophobic Au NPs, MF NFs, MF@Au NFs, MF@Au@PEI NFs and citrate capped Au NPs. (f) ζ-potential of MF NFs, hydrophobic Au NPs, MF@Au NFs and MF@Au@PEI NFs. Figure S6. High resolution (a) C 1s, (b) Mg 2p, (c) Fe 2p, and (d) O 1s spectra of MF NFs. Figure S7. MF@Au NFs coated with different concentrations of PEI (concentrations of (a–d) are 5, 1, 0.5, 0.2 mg/mL in order, the scale bar is 2 μm). Figure S8. Photographs of the detection results of different concentrations of HE4 (0, 10, 50, 100, 200, 400, and 800 pM) using citrate capped Au NPs as optical labels [45,46,47].
