# Supplementary material for: High-Density Gold Nanoparticles Implanted on Mg/Fe LDH Nanoflowers Assisted Lateral Flow Immuno-Dipstick Assay for Visual Detection of Human Epididymal Protein 4
Source: Biosensors (Basel). 2022 Sep 27;12(10):797. doi: 10.3390/bios12100797 (PMC9599355; doi:10.3390/bios12100797)
Supplement: Supplementary file 1 [file biosensors-12-00797-s001.zip › biosensors-1918105-supplementary.pdf]

Electronic Supplementary Information

High-density of Gold Nanoparticles Implanted Mg/Fe LDH Nanoflowers Assisted Lateral Flow Immuno-Dipstick Assay for Visual Detection of Human epididymal protein 4

Hao **Liu**[1]**,** Mei-Xia **Wu**[2]**,** Shou-Nian **Ding**[1, *]

[1] Jiangsu Province Hi-Tech Key Laboratory for Bio-medical Research, School of Chemistry and Chemical Engineering, Southeast University, Nanjing 211189, P.R. China

[2] Lianshui Peoples Hospital, Huaian 223400, Jiangsu, Peoples R China

E-mail: snding@seu.edu.cn

Synthesis of citrate capped Au NPs

The preparation of citrate capped Au NPs with a diameter of ∼20 nm was carried out according to the reported methods with appropriate modifications [1,2]. After the 250 mL three-necked flask was cleaned with freshly prepared aqua regia solution, 1 mL of 1 % chloroauric acid solution was added, and 99 mL of pure water was mixed evenly. The temperature was raised to 130 ℃ until the solution in the system boiled vigorously, and 1.8 mL of 1 % sodium citrate solution was added dropwise, and the solution changed from dark blue to wine red within a few minutes. After cooling at room temperature, store at 4 ℃ until use.

Preparation of citrate capped Au NP-Ab_2 bioconjugates

Au NP-Ab_2 bioconjugates were prepared as previously reported [3]. First, 1.2 μg of Ab_2 was added into Au NPs solution, whose pH was adjusted to 8 under gentle mixing uniformly. After being stirred slightly for another 30 min at 25℃, the final solution was mixed with 25 μL of 5% BSA and stirred for another 30 min for blocking. Then, the products were collected by centrifuging at 1500 rpm for 20 min to remove large-particle impurity, followed by centrifuging at 15000 rpm for another 20 min. Finally, the obtained products were redispersed in 250 μL of 10 mM PBS (pH 7.4) containing 5% sucrose and 1% BSA for further use.

Detection of HE4 with the citrate capped Au NPs

Different concentrations of 60 μL HE4 standard target solutions (0, 5, 10, 50, 100, 200, 400, and 800 pM) were premixed with Au NP-Ab_2 bioconjugates (40 μL), respectively. Afterward, the mixtures were applied to the sample pad. Each concentration was detected three times. After 10-15 min, qualitative results could be obtained by observing the red bands on the strips.

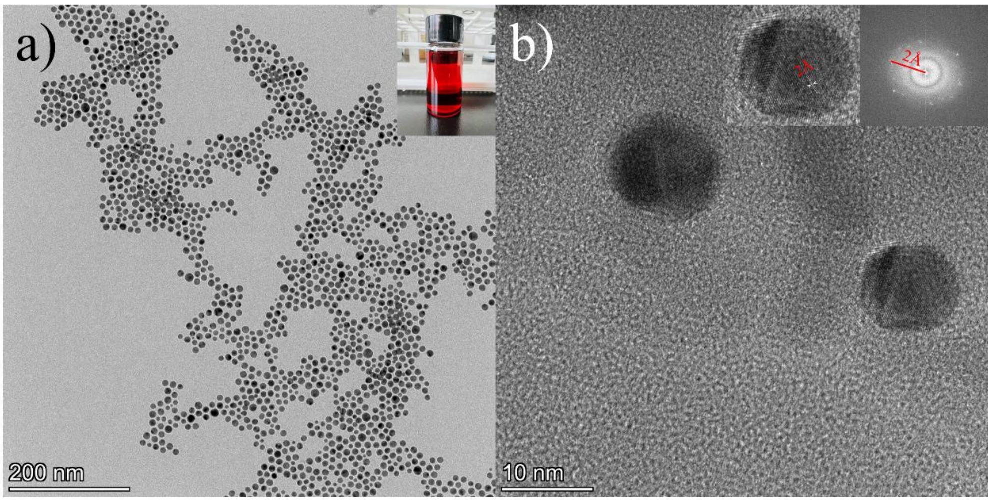

Figure S1. OLM capped Au NPs.

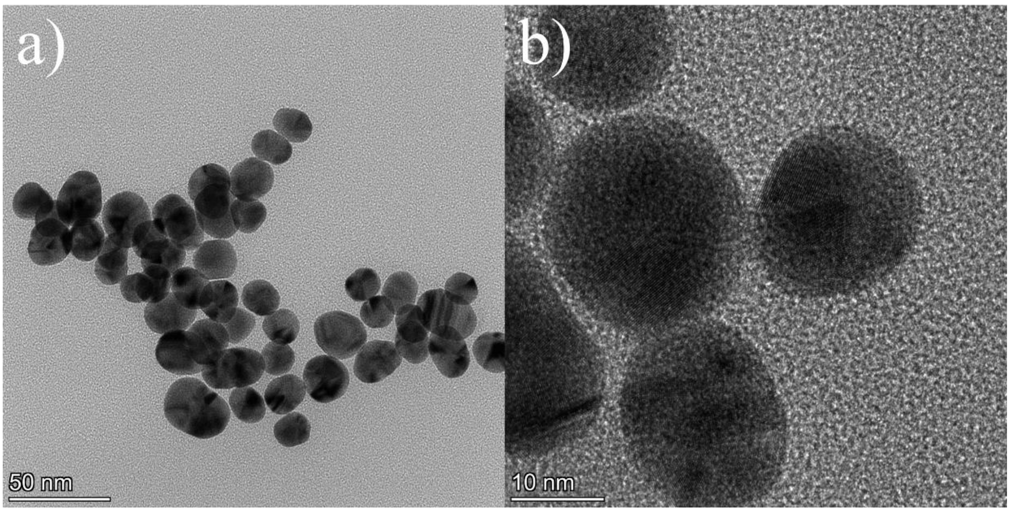

Figure S2. Citrate capped Au NPs.

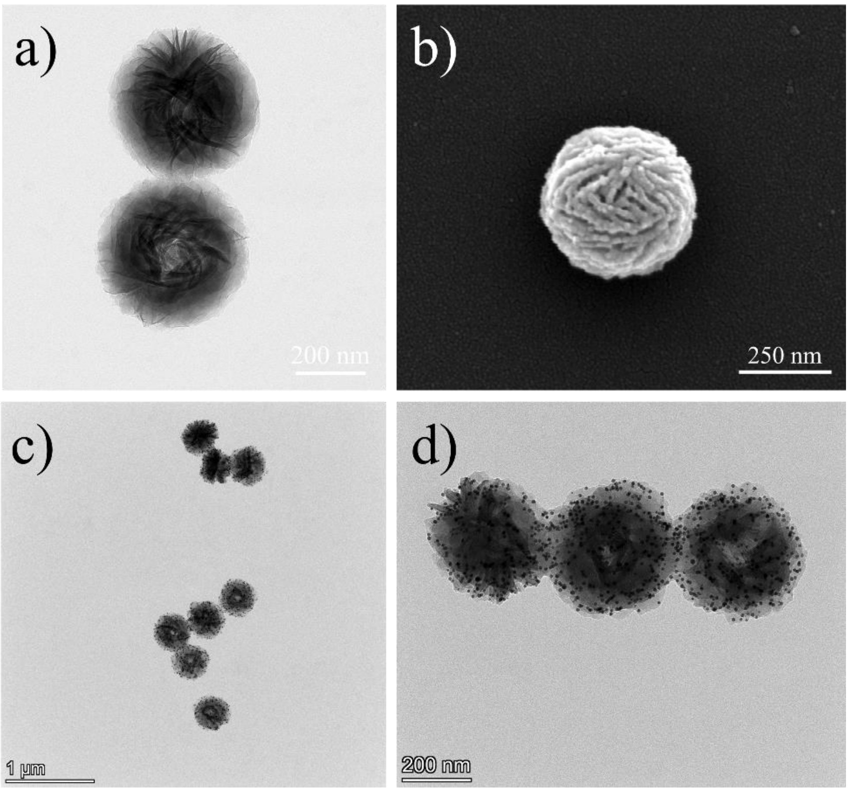

Figure S3. a). TEM image of MF NFs. b). SEM image of MF@Au NFs. c-d). TEM image of MF@Au@PEI NFs.

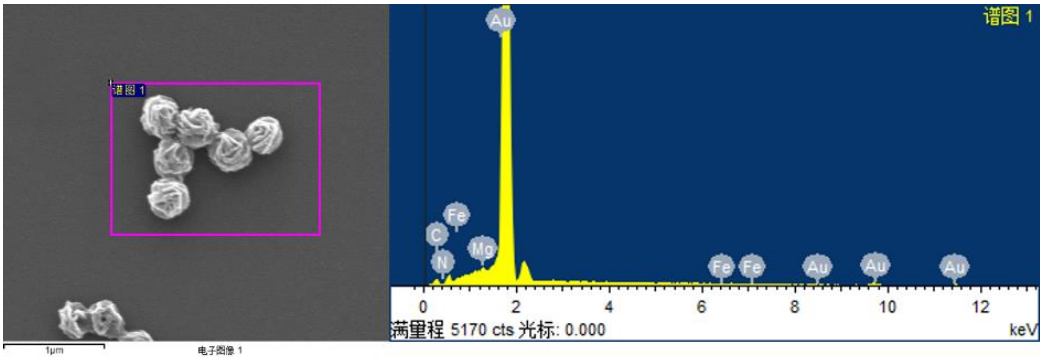

Figure S4. The EDS spectrum of MF@Au@PEI NFs.

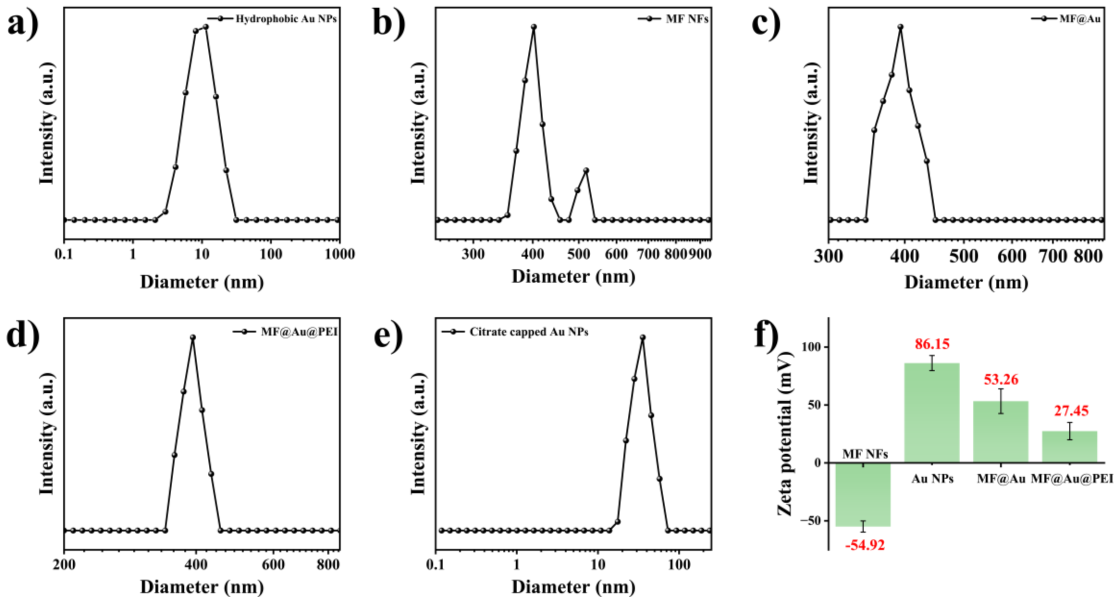

Figure S5. a-e) DLS data of hydrophobic Au NPs, MF NFs, MF@Au NFs, MF@Au@PEI NFs and citrate capped Au NPs. f) ζ-potential of MF NFs, hydrophobic Au NPs, MF@Au NFs and MF@Au@PEI NFs.

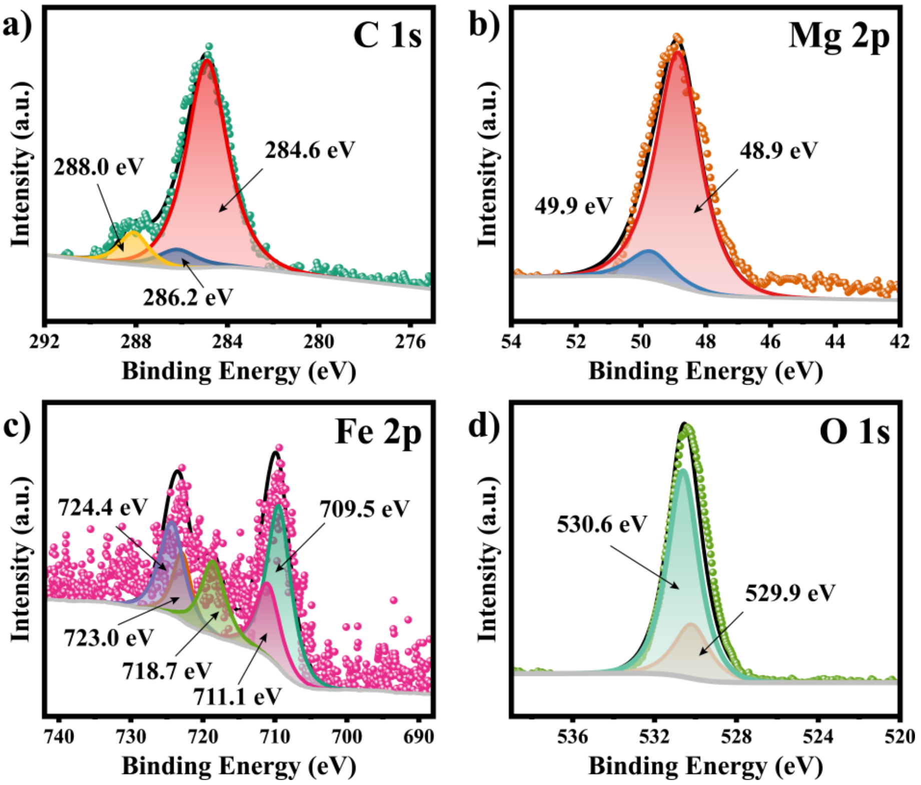

Figure S6. High resolution (a) C 1s, (b) Mg 2p, (c) Fe 2p, and (d) O 1s spectra of MF NFs.

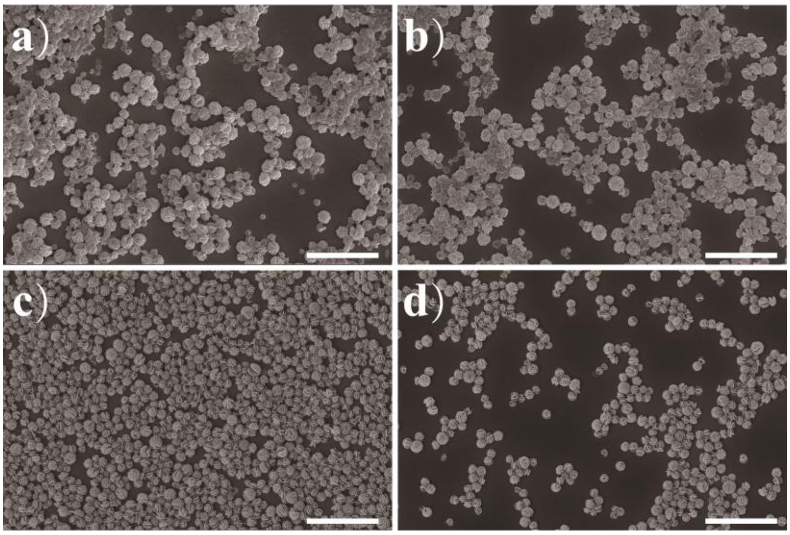

Figure S7. MF@Au NFs coated with different concentrations of PEI (concentrations of a-d are 5, 1, 0.5, 0.2 mg/mL in order, the scale bar is 2 μm).

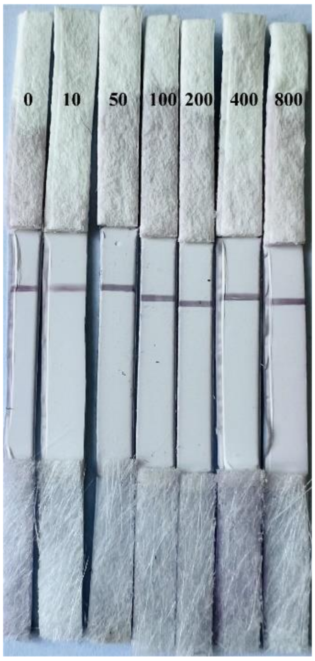

Figure S8. Photographs of the detection results of different concentrations of HE4 (0, 10, 50, 100, 200, 400, and 800 pM) using citrate capped Au NPs as optical labels.

References

1. Zhou, Y.; Pan, F.-G.; Li, Y.-S.; Zhang, Y.-Y.; Zhang, J.-H.; Lu, S.-Y.; Ren, H.-L.; Liu, Z.-S. Colloidal gold probe-based immunochromatographic assay for the rapid detection of brevetoxins in fishery product samples. *Biosensors and Bioelectronics* **2009**, *24*, 2744-2747, doi:https://doi.org/10.1016/j.bios.2009.01.034.
2. Frens, G. Controlled Nucleation for the Regulation of the Particle Size in Monodisperse Gold Suspensions. *Nature Physical Science* **1973**, *241*, 20-22, doi:10.1038/physci241020a0.
3. Zuo, J.-Y.; Jiao, Y.-J.; Zhu, J.; Ding, S.-N. Rapid Detection of Severe Fever with Thrombocytopenia Syndrome Virus via Colloidal Gold Immunochromatography Assay. *ACS Omega* **2018**, *3*, 15399-15406, doi:10.1021/acsomega.8b02366.
